# Evaluating the Dynamics of Bluetooth Low Energy Based COVID-19 Risk Estimation for Educational Institutes

**DOI:** 10.3390/s21196667

**Published:** 2021-10-07

**Authors:** Abdulah Jeza Aljohani, Junaid Shuja, Waleed Alasmary, Abdulaziz Alashaikh

**Affiliations:** 1 Department of Electrical and Computer Engineering, King Abdulaziz University, Jeddah 21589, Saudi Arabia; ajaljohani@kau.edu.sa; 2 Department of Computer Science, COMSATS University Islamabad, Abbottabad Campus, Abbottabad 22060, Pakistan; 3 Computer Engineering Department, College of Computer and Information Systems, Umm Al-Qura University, Makkah 21955, Saudi Arabia; wsasmary@uqu.edu.sa; 4 Computer Engineering and Networks Department, University of Jeddah, Jeddah 21959, Saudi Arabia; asalashaikh@uj.edu.sa

**Keywords:** BLE, machine learning, classification, COVID-19, epidemic model

## Abstract

COVID-19 tracing applications have been launched in several countries to track and control the spread of viruses. Such applications utilize Bluetooth Low Energy (BLE) transmissions, which are short range and can be used to determine infected and susceptible persons near an infected person. The COVID-19 risk estimation depends on an epidemic model for the virus behavior and Machine Learning (ML) model to classify the risk based on time series distance of the nodes that may be infected. The BLE technology enabled smartphones continuously transmit beacons and the distance is inferred from the received signal strength indicators (RSSI). The educational activities have shifted to online teaching modes due to the contagious nature of COVID-19. The government policy makers decide on education mode (online, hybrid, or physical) with little technological insight on actual risk estimates. In this study, we analyze BLE technology to debate the COVID-19 risks in university block and indoor class environments. We utilize a sigmoid based epidemic model with varying thresholds of distance to label contact data with high risk or low risk based on features such as contact duration. Further, we train multiple ML classifiers to classify a person into high risk or low risk based on labeled data of RSSI and distance. We analyze the accuracy of the ML classifiers in terms of F-score, receiver operating characteristic (ROC) curve, and confusion matrix. Lastly, we debate future research directions and limitations of this study. We complement the study with open source code so that it can be validated and further investigated.

## 1. Introduction

The COVID-19 virus has been declared a pandemic by the World Health Organization with more than 150 million cases and 3 million deaths worldwide as of 1 May 2021 [1]. The COVID-19 virus cases have increased exponentially and formed mutations indicating its highly contagious characteristics. The cure for COVID-19 can take several months due to clinical trials on animal and humans of varying ages and ethnicity before approval and possible genetic mutations shown by the virus [2]. Currently, the human community at large has no other option than to follow either containment or mitigation strategies to stop its contagious spread. Computing systems, specifically smartphones, can help in the application of containment and mitigation strategies at large [3]. Smartphones are ubiquitous. A smart application tracing COVID-19 patients can alert the community at large to avoid potentially disease infected areas. Such applications have been developed at regional/country level [4]. D2D communications, Bluetooth, and GPS technology can be applied to identify people at risk from the COVID-19 virus. As smartphones are commodity, people can use a smartphone application to be alert of their community situation regarding COVID-19 [2].

Bluetooth based indoor localization has seen renewed interest post COVID-19 outbreak [2,5]. Singapore government launched an application for tracking of COVID-19 (https://www.tracetogether.gov.sg/, accessed on 22 September 2021). The application only traces in active mode aided by Bluetooth Low Energy (BLE) technology. The smartphone users install the tracing application and turn on the Bluetooth. The application transmits beacons at constant interval to nearby devices. As a result, record of persons within proximity of few meters is maintained by each user. After a person has been tested positive for the COVID-19 virus, his smartphone transmits a record of each received beacon to a cloud database to help identify members of community at risk. The physical contacts are alerted for medical testing, isolation, and monitoring for virus symptoms. The foremost challenge to widespread deployment of such applications is that the users should trust the application to keep their private information, such as, identity and location hidden from third parties, other users of the same application, and potential hackers [6]. Several countries have launched independent contact tracing applications for COVID-19 surveillance and mitigation [7].

Educational institutes have shifted towards online teaching and hybrid teaching modes instead of on-campus mode to limit the risk of COVID-19 transmissions. Most the closures have been dictated by the government policies of lockdown and non-pharmaceutical interventions. However, there has been little technological insights on the epidemiological risk of COVID-19 that may guide the institute closure policies [8,9]. The BLE technology can be beneficial while translating the received signal strength indicator (RSSI) and distance values to risk factors and estimating the risk based on time-series mobility data. Real-time university mobility and contact data needs to be curated based on BLE technology based smartphone applications for precise evaluation of risk estimation in educational institutes. The availability of such datasets is limited. In this article, we formulate a framework for Machine Learning (ML) based COVID-19 risk estimation for educational institutes. The framework sheds light on the risk scores and the performance of five ML classifiers suitable for indoor and outdoor risk classification. We select two datasets that are were obtained in environments similar to educational institutes.

The contributions of this article are as follows,

- We predict the COVID-19 risk based on sigmoid epidemic model and five ML classifiers while utilizing two BLE datasets that depict a university outdoor and an indoor class environment. The analysis enables policy makers in determining the COVID-19 risk to students in university campuses based on contact traces and make appropriate decisions regarding the mode of teaching.
- We analyze the varying parameters, such as distance threshold, epidemic model, and classifier threshold to evaluate the COVID-19 risk in various settings for in-depth analysis.
- We make the code and pre-processed datasets open source for the extension, verification, and validation of existing research (https://github.com/jshuja86/BLE-based-COVID19-Risk-Estimation-, accessed on 22 September 2021).

The rest of the article is organized as follows. Section 2 presents the related work on BLE technology applied for COVID-19 contact tracing. Section 3 provides background knowledge on BLE contact tracing and motivation for the existing work. Section 4 details the experimental setup in terms of overall system framework, datasets, ML classifiers, and epidemic models. Section 5 presents the results for the performance evaluation of the ML classifiers in terms of ROC, F-score, and confusion matrix. The concluding remarks, limitations of existing works, and future research directions are debated in Section 6.

## 2. Related Work

Contact tracing has been utilized in containment of contagious diseases. BLE technology based contact tracing came to prominence due to the COVID-19 outbreak resulting in millions of casualties. The implementation of contact tracing applications has been very beneficial for countries with small populations. However, the efficacy seems limited for larger populations [2,10]. The foremost consideration in this case is that the participants should be aware of these risks and the scale of information shared with other users and government agencies. User should be informed of their right to refuse or withdraw their consent for use of their data. To address the user privacy issues, some efforts have been recently made as summarized in [11].

ML has been applied to the RSSI and distance values to estimate the risk of COVID-19. Most of indoor localization studies and corresponding datasets before COVID-19 measured BLE RSSI between a mobile node and multiple static receivers [12,13]. However, the COVID-19 contact tracing requires multiple mobile senders and receivers broadcasting BLE beacons in a controlled environment. The accuracy of applications for BLE based contact tracing and localization is debatable due to the variance in RSSI values based on application parameters, environment factors such as shadowing and fading, and hardware profiles [14,15,16]. However, such applications provide an efficient mechanism for automated BLE contact tracing for large population and are widely used.

The authors of [17] study BLE based contact tracing for precision, utilization, and efficiency while controlling the infections with the help of mathematical models. The authors first analyze the willingness of users, hence, percentage of population utilizing the contact tracing technology. Afterward, centralized and decentralized application models are compared to analyze the speed of contact tracing model. Using a three-state susceptible, infected, recovered (SIR) epidemiological model, the effectiveness of contact tracing is evaluated. The state transitions of the epidemic classes are modeled based on several factors, such as, reproductive number, recovery rate, transmission rate, and average quarantine period. The result show that contact tracing is not effective if no quarantine measures are taken. Moreover, contact tracing is not effective if more than 80% population does not use the application.

Felix et al. [18] proposed a risk estimation model for COVID-19 transmissions based on BLE technology. Two separate experiments were conducted with more than 40 users to obtain BLE RSSI values at Julius Leber barracks in Berlin. Each data point consists of a time series distance and RSSI values. An epidemiological model is defined that converts time series RSSI and distance values to risk binary scores. The epidemiological model is chosen from linear, box, and sigmoid functions such that increasing distance results in lower infection score. Infection scores are integrated with contact time to obtain ground truth labels. A contact time of 2 min with less than 2 m distance is considered highly infectious [19]. A linear regression model is trained to predict infection score based on the risk model. RSSI features such as sum, mean, max etc., are input to the ML model. The linear regression model based predictions strongly correlate with the linear risk model.

Pai et al. [20] proposed a two-dimensional solution named smart contact tracing enabled by BLE technology. Firstly, user contacts are classified into high or low risk based on BLE based proximate sensing. Secondly, a privacy preserving protocol is utilized for user anonymity. The authors obtain and maintain an open source BLE RSSI dataset for six positions between two users to model the RSSI variance due to body shadowing effect. Moreover, data is pre-processed with moving average of two window sizes to smooth the variance in RSS values. Five ML classifiers are employed, namely, decision tree, linear discriminant analysis, naive bayes, k-nearest neighbors, and support vector machine to classify the time series BLE RSSI values as high or low risk. Decision tree is found to be the most accurate classifier. The epidemic model considers the distance of less than two meters as high risk without taking into consideration the contact period. Fox et al. [8] analyze the impact of various public health measures including reduced class population and social distancing of 6 feet on COVID-19 transmissions at an Indiana university campus with 12,000 people. Despite strict public health measures, an outbreak was identified within first two weeks of semester. It would be very interesting to experiment with BLE contact tracing at a large university campus to evaluate the dynamics of virus propagation. However, the aforementioned study employed manual contact tracing which is very cumbersome for a large university campus.

The main issue in the listed works is the unavailability of automated BLE contact tracing data from large scale educational institutes. Such datasets are necessary to evaluate the dynamics of virus propagation in educational institutes. To address this issue, we undertook this research with multi-dimensional contributions. Firstly, we employed two datasets that closely resemble indoor class environment [20] and a university campus block [18]. Afterward, we label data with risk scores based on RSSI and distance vectors input to the epidemic (sigmoid) model. Moreover, we utilize five ML classifiers to classify risk into high and low risk categories while varying distance and classifier thresholds. Furthermore, we analyze the accuracy of the classifiers with ROC, F-score, and confusion matrix. We debate the limitations of the existing work and address future directions for smart contact tracing in educational institutes while concluding the article. Lastly, we make the datasets and code open source for validation and verification of the research work.

## 3. Background and Motivation

**Figure 1:**
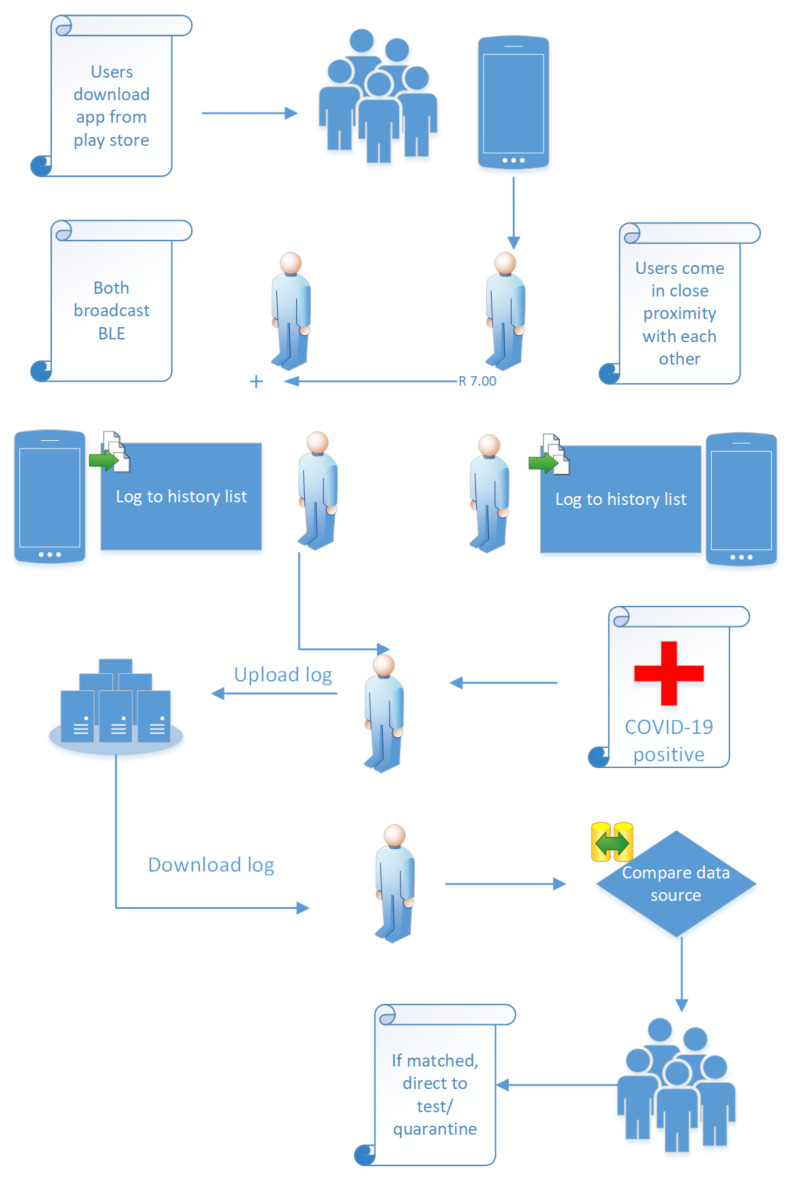
BLE based contact tracing application operation.

A BLE application works in the following manner. The application generates a pseudo-random number based on a seed and current timestamp, encrypts it with a private key, and broadcasts it in fixed time intervals. Other users in proximity listen to broadcast messages, save them in a log, and broadcast their own broadcast messages. Each smartphone keeps two records. One for the generated seeds and the other for received chirps. Testing authorities generate random permission numbers and distribute them to testing officers to authorize upload of contact information to exposure database. If a user is tested positive, his private key and generated pseudo-random numbers are uploaded to the exposure database. The information consists of multiple fields over an approximate period of two weeks where each field includes the seed used to generate chirps, the encryption keys, and the time interval between which this seed was used. Other users download the exposure database, use the user’s public key to decrypt their broadcasts, and match with the broadcasts they received to determine the extent of risk. The number of matching seeds and the signal strength indicate the physical exposure of a person and function as the measure of COVID-19 risk [21,22]. The workflow of BLE contact tracing is explained in Figure 1.

The Singapore Ministry of Health launched an app for tracking of COVID-19 [23,24]. The application only traces in active mode aided by Bluetooth. When user smartphones are in contact the devices exchange random strings as tokens. These tokens act as a user identifier for the device for a set period of time. When a user is diagnosed with COVID-19, the government requires them to upload their list of collected tokens and it is against the law not to cooperate. The application puts the government at higher control of user data with little authority to user over what details are shared. Moreover, frequent Bluetooth communications limit the battery time of the smartphones. Similar applications at government level have been developed with varying privacy mechanisms [22,25].

**Motivation:** The COVID-19 pandemic has affected normal routines of businesses, travel, and educational institutes. Educational institutes have shifted to hybrid and on-line teaching modes to limit the contacts and follow governmental lockdown policies. As a result, the quality of education has suffered due to lower concentration and attendance and where a part of student population has resided in remote areas with limited network connectivity. The shutdown of educational institutes and shift to hybrid and online teaching mode requires careful consideration of the epidemic risk [8,26]. BLE contact tracing and ML provides valuable apprehensions for policy makers to decide upon mode of teaching. Currently, the risk prediction models take the distance and time of contact between users as input. However, they can be extended to consider other epidemic parameters such as positivity rate and growth rate.

## 4. Experimental Setup

In this section, we describe and illustrate the overall research methodology, data sets, epidemic models, and ML classifiers utilized in this research work.

### 4.1. Research Methodology

**Figure 2:**
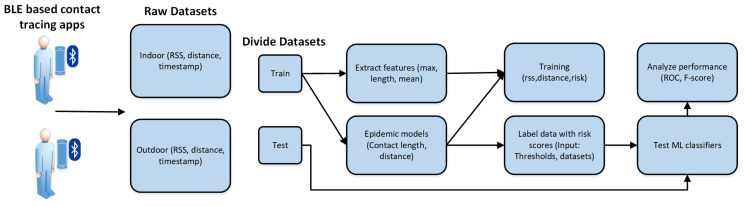
The research methodology for ML based risk prediction on BLE data.

The proposed method takes the vector of RSS values for a person and predicts whether the subject is at risk of getting infected or not. The shallow classifiers including logistic regression, decision tree, and SVM have been compared to be used as the ML models for the prediction of infection. These ML models are trained using a data set of all RSS vectors for multiple subjects with corresponding binary labels indicating the subject being at risk for the RSS value vector. The inputs to the ML models are three features of vector length, maximum, and mean while the output is the binary risk label. The risk labels corresponding to the RSS value vectors have been obtained by applying a mathematical epidemic model to the vectors of distance values corresponding to the RSS values available in the data set. The epidemic model is a mathematical function applied to the vector of distance values and resulting in a binary output. For example, the sigmoid epidemic model used for this article sums the sigmoid values for the values in the vector of distances. Sigmoid has been found to be a more effective epidemic model as compared to the linear functions for the particular data used in this research. Moreover, the classification threshold is set to then flag the predicted probabilities as at risk or safe. This study conducted the tests by varying the classification threshold, the distance reference threshold, epidemiological function, and ML model. Figure 2 illustrates the proposed research methodology.

### 4.2. Datasets

We utilized two datasets for our research. The datasets were made open source by [20] collected from an indoor classroom and [18], which is collected from military barracks resembling a university block. The original datasets can be obtained from their respective repositories (https://ieee-dataport.org/open-access/rssdatahumanhuman, https://github.com/felisat/ble-proximitiy-tracing, accessed on 22 September 2021). Data wrangling and pre-processing was required to obtain uniform features across both data sets. The first data set contains time-series scalar values of a user, the RSS, distance, and risk. The second dataset contained time-series vectors of RSS, time, and, distance representing contacts between users and their duration. We converted the scalar values to vectors so that both data sets can be simultaneously utilized for analysis. The data sets used for our research can be found at (https://github.com/jshuja86/BLE-based-COVID19-Risk-Estimation-, accessed on 22 September 2021). We are working towards a multi-user BLE dataset based on university campus (University of Jeddah) and indoor classroom to further enhance the COVID-19 risk analysis.

The first data set [20] utilized two smartphone users in different positions to obtain ground truth values for RSS, distance, timestamp, etc. The application is configured to advertise beacons at 100 ms interval with users in varying positions. A total of 13 positions and corresponding distances are measured ranging from 2 m to 5 m with each position experimented for at least 60 s. Moreover, six smartphone positions, including pocket-to-pocket (both users have smartphone in pocket) and hand-to-hand (both users have smartphone in hand), are tested between the two users to analyze the variance in RSS. As a result, the data set consists of nearly 120,000 data points. We name it the indoor dataset for naming convention in the rest of the article. The indoor dataset is arranged into different files based on position of BLE devices held by humans. To simulate the random nature of BLE positions in real world scenario, all the files have been randomly shuffled maintaining respective distance RSSI labels. Moreover, each individual file is sorted on distance between BLE devices. Dataset shuffling also arranges the RSSI and corresponding distance values randomly for classifier training. These values are then grouped together as vectors of 200 values for classifier training and evaluation.

The second data set [18] was obtained from two different experiments carried out at five (three indoor, two outdoor) locations within the Julius Leber barracks in Berlin. The two experiments consisted of 48 and 37 users, respectively. The floor area was marked as a grid to calculate the distances and record RSSI values. A video of experiments was recorded to verify the distances. Ground truth distance values were obtained from predefined user mobility pattern on the grid. Data collected from two indoor and one outdoor location was used for training while the rest was used for validation of ML classifiers. The train data set consists of 954 data points while test data set contains 468 data points detailing RSS, time, and distance vectors along with receiver and transmitter IDs. We name it the outdoor dataset.

### 4.3. Epidemic Models

**Figure 3:**
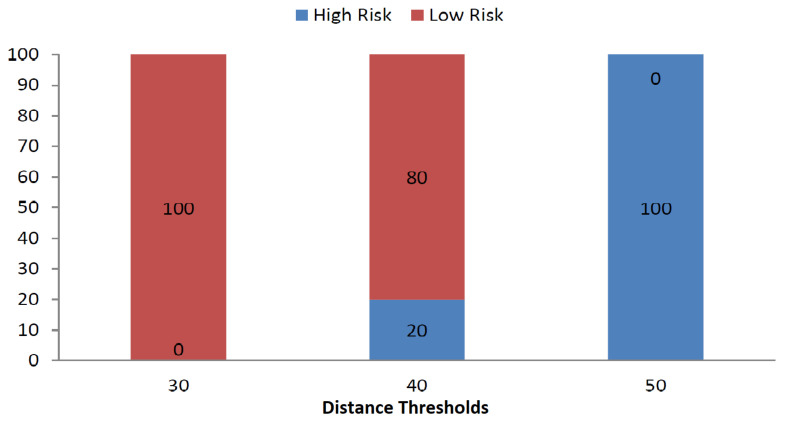
Percentage of high and low risk persons for varying thresholds: linear.

**Figure 4:**
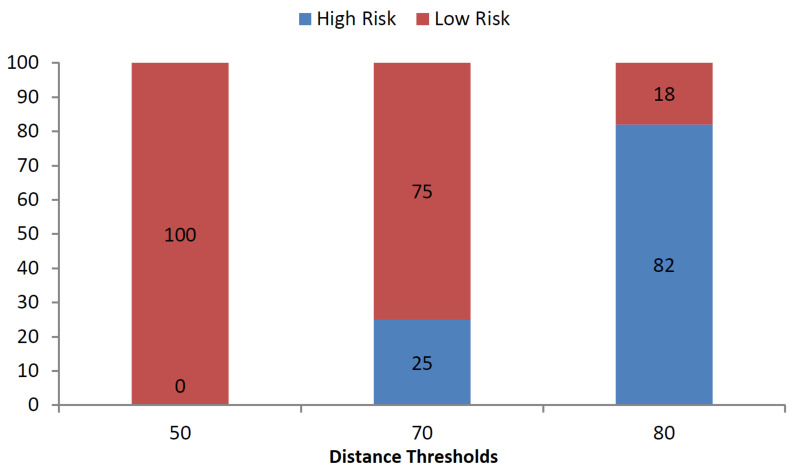
Percentage of high and low risk persons on varying thresholds: sigmoid.

The ML model is designed to predict the epidemic infection risk level as the binary classification problem using time series vectors of RSSI values by neighboring devices. An epidemic model is required to translate time series vector to risk scores. Various studies have adopted varying distance thresholds and epidemic models depending on the local Center of Disease Control (CDC) recommendations [27,28]. We can consider linear and sigmoid epidemic models for risk scores. In the sigmoid epidemic model, the sum of element wise sigmoid functions for the distance vector is compared with that of a reference distance vector and the subject is flagged as at risk based on the comparison for training the ML model. Due to the random shuffling of the indoor dataset, vectors are arranged randomly and have got marginal mean differences. As a result, linear epidemic models label all the RSSI vectors as either at risk or safe. However because of its non-linearity, sigmoid model gives us a balanced dataset in terms of risk labeling. Figure 3 and Figure 4 illustrate this fact while comparing the effect the varying threshold on risk labels for linear and sigmoid epidemic models.

The aforementioned figures show that the linear epidemic model changes the risk labels rapidly while marginally changing the distance threshold. On the contrary, the sigmoid model does not change the risk labels as quickly as compared to the linear model. Due to the change in linear model based on marginal threshold changes, all ML classifiers show similar results. As a result, we chose the sigmoid epidemic model for further analysis in this research. The Figure 3 and Figure 4 also demonstrate the percentage of students at risk of COVID-19. In the indoor environment, no students are at risk with a distance threshold of 30 cm while all students are at risk with a distance threshold of 50 cm according to linear epidemic model. The sigmoid based epidemic model labels no students at risk for 50 cm threshold while 82% students at risk for 80 cm threshold.

Epidemiological function and hence the risk flag is based on a reference vector of distances as threshold. The vector contains time series distance values where lower distance translates to high risk. Moreover, the length of vector translates to the number of contacts (higher length means higher risk). We utilized features (length, maximum, mean) of this vector for epidemic model to classify the user into risky or safe label. A reference vector is employed as threshold to train the ML model for prediction of risk scores. Both data sets cannot have the same reference vector due the heterogeneity in length and environment in which they were captured. The reference vector consists of 600 constant values of 225 cm distances measures in case of outdoor data set. The indoor data set, which is recorded inside a room, consists of RSSI vectors smaller length (<200) and distance. Moreover, the data set contains multiple values fixed distance. Therefore, we formulated a reference vector of 100 values of 75 cm distances measures. The lower values of reference vector in the indoor set are reflective of higher risks in indoor environments [29]. Increasing the reference distance threshold results in more positive labels during training. With the above reference values, sigmoid epidemic models give a balanced training set of approximately same number of low risk and high risk users.

### 4.4. ML Classifiers

Classification techniques are part of supervised ML. Multiple classification techniques and algorithms exist. We choose Support Vector machine (SVM), Decision Tree (DT), Linear Discriminant Analysis (LDA), K-Nearest Neighbors (KNN), and Logistic Regression (LR) in this article.

## 5. Results

We discuss the results with respect to the performance and validity of ML classifiers while studying effect of varying classifier (decision) threshold in this section. We chose F-score, area under the ROC (Receiver operating characteristics) curve, and confusion matrix for the performance evaluation of the ML classifiers. In case of classification tasks of biomedical applications, the classification accuracy alone is not enough for evaluation of the classifier models as false positives and false negatives have different significance. Hence ROC, F-score, and confusion matrix have been analyzed for the proposed framework for in-depth analysis. Label for each ROC curve is the AUC for that curve and classifier threshold. Label for each F-score plot represents the F-score achieved for corresponding classifier thresholds.

### 5.1. ROC

**Figure 5:**
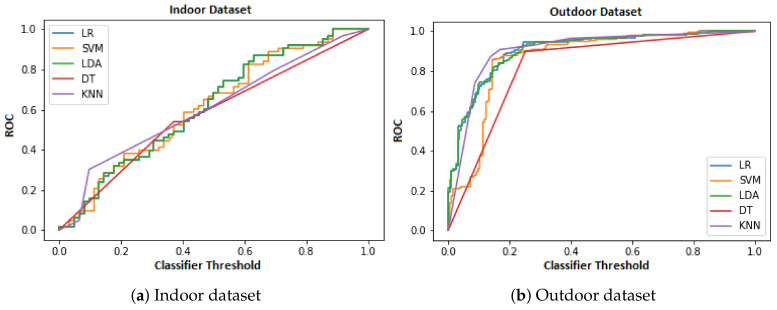
Comparison of ML classifiers for in terms of ROC.

ROC is used for binary classification tasks and plots the True Positive Rate across the False Positive Rate for various classification thresholds considered to label the sample as true positive. AUC represents area under the curve for different classification thresholds. AUC is overall performance for all thresholds. The performance (ROC) of ML classifiers for both data sets is illustrated in Figure 5a,b.

The ML classifiers are ranked as follows: LR and LDA are joint best classifiers while SVM, KNN, and DT follow for indoor datasets. For the outdoor dataset, the ML classifiers are ranked from LR, KNN, LDA, SVM, to DT. LR and KNN are the joint best ML classifier for outdoor dataset. LR is the best classifier for both data sets in terms of ROC. ML classifiers perform better on the outdoor dataset that allows for higher distance threshold and also contains contact traces of varying length and time. The ML classifiers also adjust performance quickly with increasing classifier threshold for outdoor dataset. Cumulatively, the ML classifiers perform 31% better in terms of ROC for the outdoor dataset as compared to indoor dataset. The result of ROC for both datasets in numeric form is presented in Table 1.

### 5.2. F-Score

**Figure 6:**
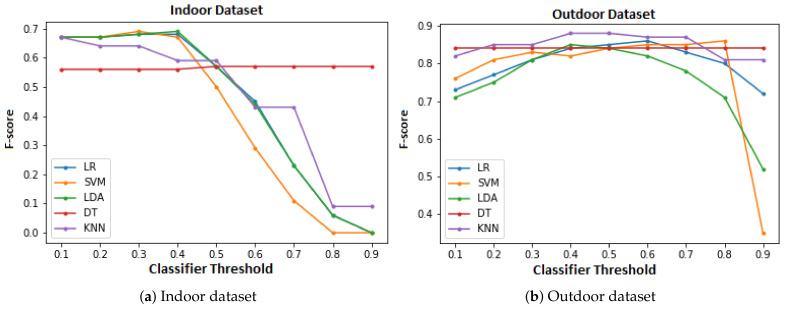
Comparison of ML classifiers in terms of F-score.

F-score is the harmonic mean of precision and recall. Harmonic mean is the representation of average for two ratios. Since precision and recall are both ratios hence their Harmonic mean, i.e., F-score is a good classification measure considering both precision and recall. Precision refers to the ratio of True Positive (TP) predictions to totally positive predictions (including False Positives (FP)), i.e., TP/(TP + FP). Recall is ratio of TP to actual positives in the dataset, i.e., sum of TP and False Negatives (FN), i.e., TP/(TP + FN). The performance (F-score) of ML classifiers for both data sets is illustrated in Figure 6a,b.

The performance of ML classifier can be interpreted for the 0.5 classifier threshold from Figure 6a. KNN provides the best F-score while DT, LDA, and LR follow for indoor dataset. SVM has the worst F-score among the analyzed ML classifiers. The DT classifier performs linearly with varying classifier threshold. The F-score of remaining ML classifiers fluctuates with varying classifier threshold. In the case of outdoor dataset, the best F-score is given by KNN while LR, DT, SVM, and LDA follow. Cumulatively, the ML classifiers perform approximately 35% better in terms of F-score for the outdoor dataset as compared to indoor dataset. Table 2 lists the best F-scores for the ML models and the corresponding classifier thresholds.

### 5.3. Confusion Matrix

**Figure 7:**
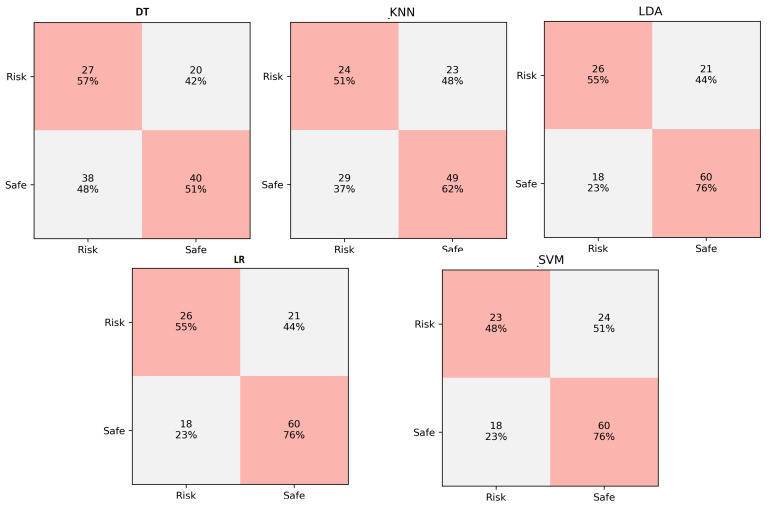
Comparison of ML classifiers for indoor dataset (confusion matrix).

**Figure 8:**
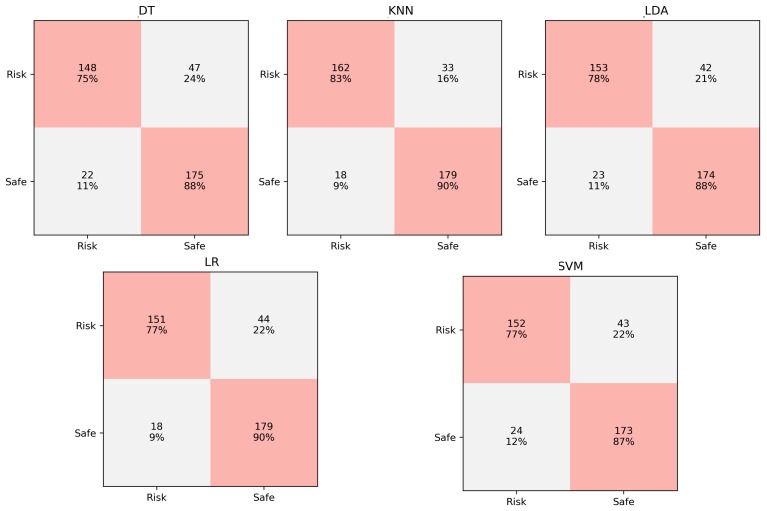
Comparison of ML classifiers for outdoor dataset (confusion matrix).

Binary classification performed by ML models can be judged based on confusion matrix if true labels are known. Confusion matrix presents the four combinations of predicted and actual values in a 2 × 2 matrix. These combinations represent true positive (TP), true negative (TN), false positive (FP, Type I error), and false negative (FN, Type II error) cases. The confusion matrix of the selected ML classification models for indoor and outdoor datasets is illustrated in Figure 7 and Figure 8, respectively.

The *x*-axis of Figure 7 and Figure 8 represent the ground truth labels while the *y*-axis represents the predicted labels. The top left cell shows the number and percentage of TP while the top right cells shows the number of FP. similarly, the bottom right cell shows the number and percentage of FN while the bottom right cell represents TN. The results for the indoor dataset show that the number of errors (type I and II) is lowest for LDA and LR classifiers while the DT classifier has the highest errors. The result of the outdoor dataset shows best performance in terms of low errors is achieved by the KNN classifier while DT is the worst classifier.

## 6. Conclusions, Limitations, and Future Directions

BLE contact tracing is an essential part of COVID-19 mitigation and a non-pharmaceutical measure to stop the virus outbreak. BLE traces can be used to predict the virus transmission risk based on epidemic models and ML classifiers that classify RSSI and contact duration in risk scores. We analyzed two datasets resembling university indoor and outdoor environments to get insights on COVID-19 transmission risks among students. We employed sigmoid based epidemic model and calibrated the thresholds to balance the risk scores. Multiple ML classifiers were analyzed for performance based on F-score, ROC, and confusion matrix. We found that LR classifier performs best in terms of ROC for both data sets. Moreover, KNN is the best classifier in terms of F-score for both indoor and outdoor dataset. LDA and LR classifiers have lowest errors for the indoor dataset while KNN has the lowest errors for the outdoor dataset. We complimented our research with open source data sets and code for validation and extension. The analysis of the epidemic model shows that 100% students are at risk for the indoor data set at 50 cm threshold in case of linear epidemic model while no student is at risk for the same threshold in case of sigmoid epidemic model.

**Limitations:** There are several limitations regarding existing BLE based contact tracing research. They are listed as bellow,

- The employed datasets resemble an university classroom and block environment but do not ideally represent it. For example, the data set presented in [20] consists of only two students in a classroom. Real-time datasets collected from universities are necessary to analyze the corresponding risk factors. Moreover, such datasets can help analyze multiple hypotheses such as: If social distancing guidelines are followed, is it possible to eliminate COVID-19 risk in a university campus? What if the students are divided into two shifts to decrease class densities?
- The efficacy of any risk estimation based on contact tracing technology is limited to the application utilization. The utilization of contact tracing applications even among smaller and developed populations, such as Singapore is as low as 20%. As a result, only 4% of the contacts will be traced limiting the efficacy of risk estimation that is solely based on BLE technology [17].
- Contact tracing applications can be categorized into centralized versus decentralized database models. The centralized model automatically allows health authorities to utilize the contact history of infected person for fast action. However, the users have privacy concerns as centralized model can result in mass population surveillance. Decentralized model gives the contact update authority to the user lowering privacy concerns while slowing the pace of contact tracing due to user unwillingness to share data [17,30].
- Training set labels and corresponding classifier are heavily dependent on the epidemic model and threshold vector used for the epidemic model. Slight changes in the reference distance vector length or values result in imbalanced datasets and effect the classification model significantly. Hence, the epidemic model and the distance vector reference need to be selected carefully considering local dynamics and mathematical epidemiology [31].

**Future directions:** This work provides a baseline for several future directions enabled by the open source code. These are listed below.

- The existing classifiers employed for risk classification are binary. More precise epidemic models should be investigated to classify users into infected, susceptible, and at risk classes.
- The existing datasets contained limited number of users while an educational institute can have thousands of users with BLE enabled smartphones. The application of ML classifiers on large datasets necessitates evaluation of time and memory consumption for the ML classifiers to identify resource efficiency [32].
- The validation of epidemic models is necessary with the help of a dataset obtained from a bio-secure environment so that the dataset includes the COVID-19 PCR test results. Consequently, we will be able to validate whether the epidemic model is providing the same virus propagation results as the PCR test results indicate?

## Figures and Tables

**Table 1:** Comparison of ROC.

Datasets	ML Model				
	LR	SVM	LDA	DT	KNN
Indoor	0.62	0.61	0.62	0.58	0.61
Outdoor	0.91	0.87	0.90	0.82	0.91

**Table 2:** Comparison of Best F-scores.

Datasets	ML Model (Classifier Threshold)				
	LR	SVM	LDA	DT	KNN
Indoor	0.68 (0.3)	0.69 (0.3)	0.69 (0.4)	0.57 (0.5)	0.67 (0.1)
Outdoor	0.86 (0.6)	0.86 (0.8)	0.85 (0.4)	0.84 (0.1)	0.88 (0.4)

## Data Availability

The data and the code can be found at https://github.com/jshuja86/BLE-based-COVID19-Risk-Estimation-, accessed on 22 September 2021.
